# Supplementary material for: Pharmacokinetic Modeling of Hydrocortisone by Including Protein Binding to Corticosteroid-Binding Globulin
Source: Pharmaceutics. 2022 May 30;14(6):1161. doi: 10.3390/pharmaceutics14061161 (PMC9231005; doi:10.3390/pharmaceutics14061161)
Supplement: Supplementary file 1 [file pharmaceutics-14-01161-s001.zip › pharmaceutics-1681893-supplementary.pdf]

Supplementary S1

```
Berkeley Madonna Script (version 8.3)
{ Code generated by Edsim++ }
METHOD RK4
STARTTIME = 0
STOPTIME = 30
DT = 0.01 {fixed}
DISPLAY C01_C,C01_Cu,fu_Var
{ Parameter Initialization }
P01_Age = 55
P01_Bw = 70
C01_CBG = 44
C01_ALB = 44555
C01_Kca = 21945
C01_Kci = 1.815
C01_Kce = 18.15
C01_RC = 0.1
C01_V = 474.38
PO_k = 1.4
ME_CL = 235.78
{ Variable Initialization }
INIT C01_A = 0
INIT PO_A = 0
INIT AUCU_AUC = 0
INIT AUC_AUC = 0
{ Standard Equations }
Age = P01_Age + TIME/365.0/24.0
Bw = P01_Bw
C01_Cu = C01_A/C01_V
MW_ALB = 66500
MW_CBG = 55000
MW_COR = 362.46
F = C01_Cu /MW_COR * 1E6
TA = C01_ALB/MW_ALB * 1E6
TC = C01_CBG/MW_CBG * 1E6
TCI = (1 − C01_RC) * TC
TCE = C01_RC * TC;
KA = C01_Kca/MW_ALB * 1E6
KCI = C01_Kci/MW_CBG * 1E6
KCE = C01_Kce/MW_CBG * 1E6
TF = F * (1 + TA/(KA + F) + TCI/(KCI + F) + TCE/(KCE + F))
C01_fu = IF TF <> 0 THEN F/TF ELSE 1
C01_C = C01_Cu/C01_fu
PO_D = Pulse(10 * 0.96, 0, 6) − Pulse(10 * 0.96, 0 + 3 * 6, 6)
PO_R = PO_k * PO_A
ME_k = ME_CL/C01_V
ME_R = ME_k * C01_A
fu_Var = C01_fu
{ Differential Equations }
C01_A′ = +PO_R + -ME_R
```

PO_A′ = -PO_R + PO_D
AUCU_AUC′ = C01_Cu
AUC_AUC′ = C01_C

Supplementary S2

RxODE Script (version 0.7)

```
################################
#                              #
# Code generated by Edsim++ #
#                              #
################################
# R: https://cran.r-project.org/
# Rtools: https://cran.r-project.org/bin/windows/Rtools/
# RxODE: https://cran.r-project.org/web/packages/RxODE/index.html
# nlmixr: https://cran.r-project.org/web/packages/nlmixr/index.html
# Load RxODE (requires RTools)
Library (RxODE)
# Start of model (C-Language)
ode = "
# Standard Equations
Age = P01_Age + t/365.0/24.0;
Bw = P01_Bw;
C01_Cu = C01_A/C01_V;
MW_ALB = 66500;
MW_CBG = 55000;
MW_COR = 362.46;
F = C01_Cu /MW_COR * 1E6;
TA = C01_ALB/MW_ALB * 1E6;
TC = C01_CBG/MW_CBG * 1E6;
TCI = (1 − C01_RC) * TC;
TCE = C01_RC * TC;
KA = C01_Kca/MW_ALB * 1E6;
KCI = C01_Kci/MW_CBG * 1E6;
KCE = C01_Kce/MW_CBG * 1E6;
TF = F * (1 + TA/(KA + F) + TCI/(KCI + F) + TCE/(KCE + F));
C01_fu = F/TF;
C01_C = C01_Cu/C01_fu;
PO_R = PO_k * PO_A;
ME_k = ME_CL/C01_V;
ME_R = ME_k * C01_A;
fu_Var = C01_fu;
# Differential Equations
d/dt (C01_A) = (+PO_R) + (-ME_R);
d/dt (PO_A) = (-PO_R);
d/dt (AUCU_AUC) = C01_Cu;
d/dt (AUC_AUC) = C01_C;
"
# End of model
# Compile the model
mod = RxODE(model = ode, modName = "mod")
# Initialize parameters
```

```
parm = c(P01_Age = 55,P01_Bw = 70,C01_CBG = 44,C01_ALB = 44555,C01_Kca =
21945,C01_Kci = 1.815,C01_Kce = 18.15,C01_RC = 0.1,C01_V = 474.38,PO_k = 1.4,ME_CL
= 235.78)
# Initialize variables
vini = c(C01_A = 0,PO_A = 0,AUCU_AUC = 0,AUC_AUC = 0)
# Create event table
ev = eventTable(amount.units='mg', time.units='hours')
# Set output range
ev$add.sampling(seq(0, 30, by=0.1))
# Add dosing events (all code in objects starting with ~ is appended and expanded here
after)
ev$add.dosing(dose=10 * 0.96, start.time=0, nbr.doses=3, dosing.interval=6,
dosing.to=which (vdif=="PO_A"))
# Execute simulation
result = mod$run(parm, ev, vini)
# Save results (remove comment and change path)
# write.csv(result,'result.csv')
# View results table
View(result)
```

Supplementary S3

Script Symbols

P01_Age	Age (hours)
P01_Bw	Body weight (kg)
C01_A	Amount of hydrocortisone (mg)
C01_C	Total concentration of hydrocortisone (see also TF, mg/L)
C01_Cu	Free concentration of hydrocortisone (see also F, mg/L)
C01_fu	Fraction hydrocortisone unbound (Cu/C = F/TF)
C01_V	Volume of distribution of hydrocortisone (L)
ME_CL	Clearance of hydrocortisone (L/h)
ME_R	Elimination rate of hydrocortisone (mg/h)
PO_k	Fractional absorption rate of hydrocortisone (1/h)
PO_R	Absolute absorption rate of hydrocortisone (mg/h)
PO_D	Oral dose of hydrocortisone (mg)
AUC_AUC	Area under the total hydrocortisone plasma curve (mg.h/L)
AUC_AUCU	Area under the free hydrocortisone plasma curve (mg.h/L)
C01_CBG	Corticosteroid binding globulin concentration (mg/L)
C01_ALB	Serum albumin (mg/L)
C01_Kca	Protein equilibrium dissociation constant (hydrocortisone-albumin, mg/L)
C01_Kci	Protein equilibrium dissociation constant (hydrocortisone-iCBG, mg/L)
C01_Kce	Protein equilibrium dissociation constant (hydrocortisone-eCBG, mg/L)
C01_RC	Ratio eCBG/CBG
MW_COR	Molecular weight hydrocortisone (g/mol)
MW_ALB	Molecular weight albumin (g/mol)
MW_CBG	Molecular weight CBG (g/mol)
F	Free concentration of hydrocortisone (see also C01_Cu, nM)
TF	Total concentration of hydrocortisone (see also C01_C, nM)
TA	Total concentration of albumin (nM)
TC	Total concentration of CBG (iCBG + eCBG, nM)
TCI	Total concentration of iCBG (low affinity state, nM)
TCE	Total concentration of eCBG (high affinity state, nM)

KA	Protein equilibrium dissociation constant (hydrocortisone-albumin, nM)
KCI	Protein equilibrium dissociation constant (hydrocortisone-iCBG, nM)
KCE	Protein equilibrium dissociation constant (hydrocortisone-eCBG, nM)

Supplementary S4

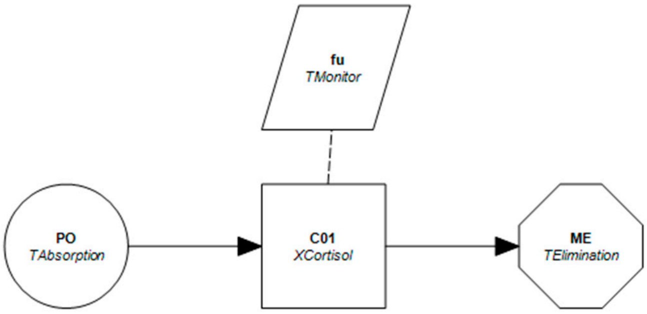

Figure S1. Model used in Edsim++ v1.7.03. One-compartment model with first-order absorption (PO object) and first-order elimination (ME object). Protein binding of hydrocortisone was modeled using a specialized central compartment (C01 object of type XHydrocortisone) that implements binding Equation (1). The model was validated by creating alternative implementations in R using the RxODE package (Supplementary S1) and in Berkeley Madonna (Supplementary S1).

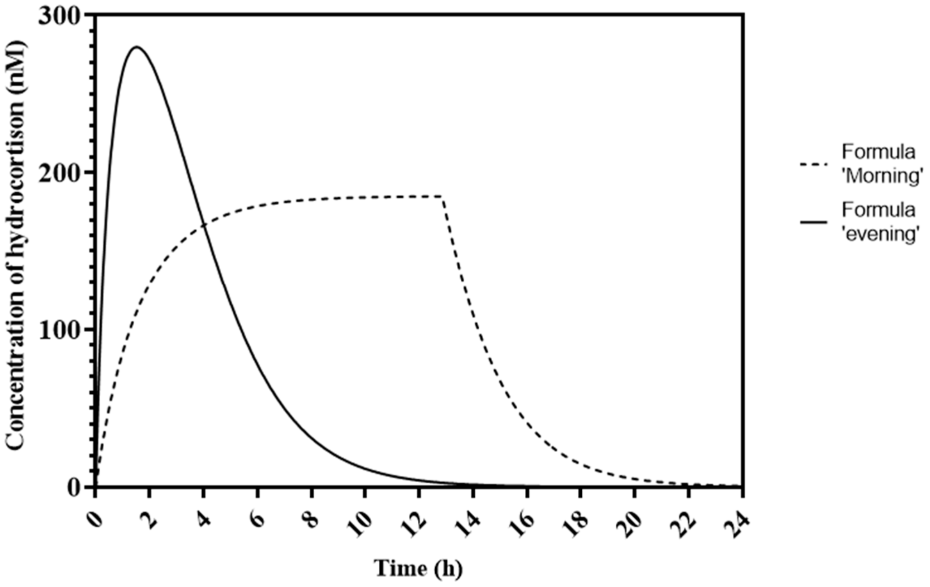

Figure S2. Formulations simulated in Edsim++. Dose is adjusted to 9 mg for evening and 5 mg for morning for this patient (weight; 70 kg, height; 1.75 m). Lag time of approximately 4 h was used. To ensure slow absorption of morning dose, zero-order elimination is 0.8 mg/h (normal value 1.0). For the evening formulation a fractional absorption rate of 0.7 L/h was chosen.
